# Cellulose Nanocrystal-Based Pickering Emulsions as Advanced Biomaterials for Food Bioactive Delivery: Chemical Modification, Synergistic Stabilization, and Functional Applications

**DOI:** 10.3390/foods15132286

**Published:** 2026-06-25

**Authors:** Haochen Ni, Kairu Li, Jiaqi Li, Suyu Li, Haoran Bai, Wenjing Dong, Fuqiang Zhang, Xinxin Yan, Jiaqi Guo

**Affiliations:** State Key Laboratory for Development and Utilization of Forest Food Resources and Co-Innovation Center for Efficient Processing and Utilization of Forest Products, Nanjing Forestry University, Nanjing 210037, China; 2351501211@njfu.edu.cn (H.N.); 2411510309@njfu.edu.cn (K.L.); ljq@njfu.edu.cn (J.L.); 1145067442@njfu.edu.cn (S.L.); 2411501301@njfu.edu.cn (H.B.); 2411501204@njfu.edu.cn (W.D.); 2411510225@njfu.edu.cn (F.Z.)

**Keywords:** cellulose nanocrystals, pickering emulsions, chemical modification, synergistic stabilization, functional applications

## Abstract

Cellulose nanocrystals (CNCs) are renewable and biodegradable nanomaterials that can stabilize Pickering emulsions through steric hindrance and electrostatic repulsion. However, pristine CNCs show limited interfacial anchoring because of their strong hydrophilicity and high surface charge density, making the emulsions susceptible to coalescence, phase separation, and structural instability under environmental stresses. This review summarizes two major strategies for stabilizing and functionally regulating CNC-based Pickering emulsions: chemical modification and synergistic stabilization. Chemical modification regulates CNC surface charge, wettability, interfacial anchoring, and functional group composition through oxidation, amination, esterification, graft copolymerization, desulfation, and etherification, whereas synergistic stabilization constructs composite interfacial films or continuous-phase networks through noncovalent interactions between CNCs and proteins, polysaccharides, cyclodextrins, surfactants, inorganic nanomaterials, or functional molecules. The ability of these emulsion systems to compartmentalize oil-soluble bioactives within structured droplets also provides a basis for improving bioactive stability and release behavior in food-related formulations. These strategies improve emulsion stability and introduce antibacterial, antioxidant, responsive, and controlled-release properties, highlighting the potential of CNC-based Pickering emulsions in active food systems, including food preservation, active packaging, and the stabilization, protection, and release regulation of food bioactives. Remaining challenges in green preparation, structural regulation, release mechanisms, scalable production, and practical evaluation are also discussed.

## 1. Introduction

Pickering emulsions have attracted increasing attention in food systems because they can stabilize hydrophobic or volatile bioactive compounds without relying heavily on conventional molecular surfactants. By adsorbing solid particles at the oil–water interface, Pickering emulsions form a protective interfacial barrier around dispersed droplets, which can reduce coalescence, improve storage stability, and protect sensitive compounds from oxidation, volatilization, or degradation during processing and storage [1]. These characteristics make particle-stabilized emulsions promising for food preservation, active packaging, nutraceutical delivery, and controlled release of food bioactives. In these food-oriented applications, emulsion performance is closely related to physical stability, interfacial properties, and delivery efficiency. Therefore, parameters such as droplet-size distribution, creaming or sedimentation index, coalescence resistance, rheological stability, resistance to pH, ionic strength, and thermal processing, particle wettability, contact angle, interfacial coverage, zeta potential, encapsulation efficiency, bioactive retention, gastrointestinal release behavior, and bioaccessibility are commonly used to evaluate whether a Pickering emulsion can function effectively as a food bioactive delivery system [2].

Cellulose nanocrystals (CNCs) are anisotropic rod-like nanoparticles that are commonly prepared by sulfuric acid hydrolysis to remove the amorphous regions of native cellulose. CNCs can be obtained from diverse raw materials and possess high crystallinity, large aspect ratios, and partial amphiphilicity, enabling them to form dense and stable particle interfacial layers at oil–water interfaces; they were reported as early as 2011 for stabilizing oil–water interfaces [3]. CNCs mainly stabilize Pickering emulsions through interfacial adsorption, electrostatic repulsion, and steric hindrance between droplets. Owing to their renewable origin, high aspect ratio, surface tunability, and ability to reinforce interfacial layers, CNCs have become promising particle stabilizers for food-related Pickering emulsions. Food-relevant biopolymers, such as alginate, gelatin, starch, pectin, chitosan, caseinate, whey proteins, and zein, have been widely used to construct gels, films, capsules, and emulsion-based delivery systems because of their good compatibility with food matrices and their ability to form networks through electrostatic interactions, hydrogen bonding, hydrophobic association, and protein–polysaccharide complexation [4,5]. Compared with these flexible or semi-flexible biopolymers, CNCs provide a rigid rod-like architecture, high crystallinity, high specific surface area, and abundant surface hydroxyl groups that can be further modified or combined with other food-grade components. These characteristics allow CNCs to act as structural and interfacial regulators by strengthening the particle layer at oil–water interfaces, improving barrier formation around droplets, reducing the need for conventional molecular surfactants, and regulating the retention and release of encapsulated bioactives. Therefore, CNCs do not simply replace traditional food biopolymers; rather, they can complement protein- and polysaccharide-based matrices by improving interfacial robustness, surface tunability, and delivery performance in food-related Pickering emulsions.

At present, CNCs derived from various biomass resources, such as lemon seeds, maple leaves, vegetable residues, corn stover, wood pulp, and rice straw, have been used to construct Pickering emulsions and have displayed diverse application value in food preservation, active compound delivery, functional coatings, and active packaging (Table 1). These studies indicate that CNCs are not only widely available renewable interfacial stabilizing particles but also provide an important pathway for the high-value utilization of biomass resources. In food preservation, CNC-stabilized Pickering emulsions can be directly coated onto food surfaces to extend shelf life, incorporated into film matrices such as gelatin to prepare composite antibacterial films with improved mechanical properties and barrier performance, or used to achieve non-contact antibacterial preservation through the volatilization of active compounds from the oil phase [6]. From a food science perspective, these emulsion systems are particularly relevant to the stabilization, protection, and release regulation of food bioactives, especially hydrophobic or volatile compounds that are easily degraded, oxidized, or lost during processing and storage. The interfacial layer and continuous-phase structure of CNC-based Pickering emulsions provide a biomaterial platform for improving the dispersion stability and functional performance of these compounds, suggesting their potential value in active food systems and bioactive delivery formulations.

However, the use of pristine CNCs to stabilize Pickering emulsions still has several limitations. The abundant hydroxyl groups on CNC surfaces impart strong hydrophilicity and weak hydrophobicity. This surface characteristic restricts the effective adsorption of CNCs at oil–water interfaces, which not only weakens their emulsifying ability but also makes the emulsions difficult to maintain stable under extreme conditions such as strong acidity and high temperature, leading to droplet aggregation [7]. In addition, CNCs prepared by sulfuric acid hydrolysis contain negatively charged sulfate ester groups on their surfaces. When used alone to stabilize emulsions, excessively strong electrostatic repulsion and steric effects may instead hinder the effective adsorption of CNCs at oil–water interfaces, weaken their emulsifying ability, and even prevent the formation of stable emulsions [8]. Therefore, improving the interfacial anchoring ability, environmental adaptability, and functional expandability of CNCs while retaining their green and renewable advantages has become a key issue for the further development of CNC-based Pickering emulsions.

To address the shortcomings of pristine CNC stabilized Pickering emulsions, current studies have mainly adopted two strategies. The first is chemical modification, in which CNCs are treated by oxidation, esterification, etherification, amidation, and other modification methods to precisely regulate their wettability and interfacial adsorption ability, enhance the adsorption of CNC particles at oil–water interfaces, and improve the coalescence resistance of Pickering emulsions under extreme environments [9,10]. Meanwhile, CNC modification can introduce specific groups, such as antibacterial groups and pH responsive groups, thereby endowing emulsions with multiple functions including antibacterial activity, antioxidant activity, and pH responsiveness [11]. The second is synergistic stabilization, which involves constructing synergistic systems composed of CNCs and components such as proteins, carbohydrates, inorganic particles, and polyphenols [7]. In this review, synergistic stabilization refers to the cooperative stabilization achieved by CNCs together with other components through interfacial adsorption, electrostatic interactions, hydrogen bonding, hydrophobic interactions, steric hindrance, and continuous-phase network formation. These components form denser composite interfacial layers through electrostatic adsorption, hydrogen bonding, and hydrophobic interactions. On the one hand, synergistic interactions can regulate the steric hindrance and electrostatic repulsion between droplets, thereby improving the coalescence and sedimentation stability of emulsions. On the other hand, the diversified combination of synergistic components can also impart multiple functions such as antibacterial and antioxidant activities to the emulsions [12].

In recent years, many reviews have focused on the sources and extraction of CNCs, emulsion preparation methods, the specific factors affecting the stability of CNC-based Pickering emulsions, such as droplet size and zeta potential, and the applications of these emulsions in food related fields. Rahmi et al. summarized the factors affecting the stability of CNC-based Pickering emulsions and the effects of these emulsions on packaging properties, including the mechanical performance and ultraviolet blocking ability of films [11]. Ji & Wang listed important applications of CNC-based Pickering emulsions in food related fields, such as active compound delivery [13]. However, most existing reviews have not fully considered the limitations of directly using CNCs to stabilize Pickering emulsions, and effective strategies for regulating emulsion stability and functionality at the mechanistic level remain insufficiently discussed. Yan et al. reviewed the specific application scenarios of pristine CNC stabilized Pickering emulsions, but further exploration of strategies for improving emulsion stability and functionality remains limited [6]. Although several reviews have discussed the chemical modification and synergistic stabilization of CNCs, these two approaches are often treated as separate research branches rather than being integrated as common strategies for improving the stability and functionality of Pickering emulsions [7,9]. Comparative discussions among different modification methods or synergistic systems are also rare. To address these gaps, this review focuses on how the limitations of pristine CNCs can be overcome through chemical modification and multicomponent synergistic stabilization. The added value of this review lies in integrating these two routes as interconnected strategies for regulating CNC wettability, interfacial adsorption, droplet stability, environmental adaptability, and functional performance. Compared with previous reviews that mainly describe CNC sources, emulsion preparation, stability factors, or application examples, this review further compares how different modification methods and synergistic systems affect interfacial structure, functionalization, and food-related application potential, while also discussing the boundaries between food-relevant systems and model material systems.

**Figure 1:**
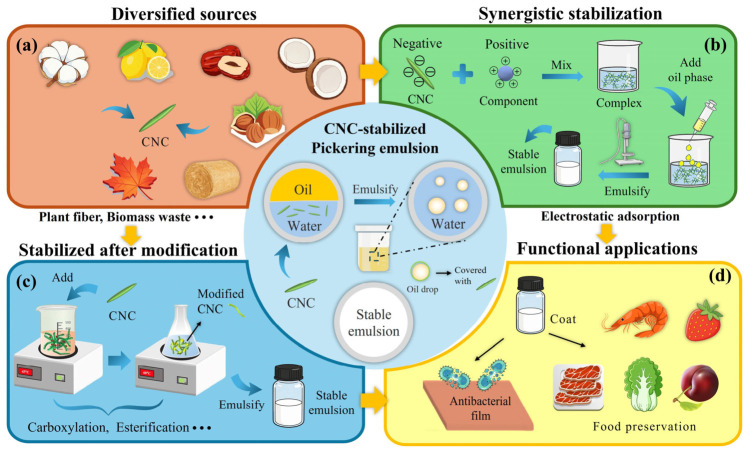
Process design and representative applications of CNC-stabilized Pickering emulsions. (**a**) Diverse sources of CNCs, including plant fibers and biomass waste. (**b**) Schematic illustration of the preparation of Pickering emulsions synergistically stabilized by CNCs and cationic agents. (**c**) Schematic illustration of the preparation of Pickering emulsions stabilized by modified CNCs. (**d**) Representative applications of CNC-stabilized Pickering emulsions involving food preservation, active packaging, antibacterial films, edible coatings, and food bioactive delivery systems.

Specifically, this review first summarizes chemical modification strategies for CNCs, including common methods such as carboxylation and esterification, and discusses their effects on CNC surface properties, interfacial adsorption ability, emulsion stability, and functional characteristics. It then outlines typical forms of synergistic stabilization systems constructed by CNCs with proteins, polysaccharides, inorganic particles, polyphenols, and other components, and analyzes their interfacial stabilization mechanisms and performance regulation effects based on microstructural characterization. On this basis, representative applications of CNC-based Pickering emulsions in food preservation, active packaging, nutraceutical delivery, and related carrier systems are further introduced (Figure 1). Particular attention is given to how CNC-based interfaces, droplet structures, and continuous-phase networks contribute to the stabilization, protection, and release regulation of bioactive compounds. This review aims to provide a systematic reference for the stability regulation, functional construction, and application expansion of CNC-based Pickering emulsions, with particular attention to their potential roles in food preservation, active packaging, and the delivery of food bioactives, and to discuss the challenges and future prospects in this field.

This review is based on a broad survey of published studies related to CNC-based Pickering emulsions, with particular emphasis on chemical modification, synergistic stabilization, interfacial regulation, and food-related functional applications. The literature was collected primarily from Web of Science, Scopus, and Google Scholar using combinations of keywords such as “cellulose nanocrystal”, “CNC”, “Pickering emulsion”, “chemical modification”, “surface modification”, “synergistic stabilization”, “protein”, “polysaccharide”, “polyphenol”, “food preservation”, “active packaging”, “bioactive delivery”, and “controlled release”. The selected literature mainly covered studies published from 2011 to 2025, with emphasis on recent studies from the past decade. In the selection process, priority was given to studies that reported CNC-based or modified CNC-based Pickering emulsions with clear information on interfacial stabilization, emulsion performance, functional regulation, or food-related application potential.

**Table 1:** Stability and functionality of Pickering emulsion stabilized by CNCs derived from biomass.

Cellulose Source	CNC Content	Organic Phase	Storage Duration	Application	Reference
Lemon seeds	0.5–1.0 wt%	Sunflower oil	14 d at 25 °C	Emulsion stabilization	[14]
Maple leaves	1.0 *w*/*v*%	Canola oil (with cinnamaldehyde)	28 d at 25 °C	Food preservation	[15]
Maple leaves	1.0 *w*/*v*%	Canola oil (with cinnamaldehyde)	28 d at r.t.	Active substance delivery	[16]
Tomato stems, Eggplant stems, Pepper stems	0.05–1.5 *w*/*v*%	Corn germ oil	30–90 d	Food preservation	[17]
Corn stover	1.5 wt%	Medium-chain triglyceride oil	90 d at r.t.	Active substance delivery	[18]
Chlorella vulgaris	0.25 *w*/*v*%	Zanthoxylum bungeanum essential oil	Not reported	Food preservation	[19]
Cotton fibers	0.3 *w*/*v*%	Oregano essential oil, Cinnamon essential oil	Not reported	Food preservation	[20]
Eucalyptus pulp	0.5–1 *w*/*v*%	Sesamol-enriched sesame oil	21 d at 26–29 °C	Active substance delivery	[21]
Wood pulp	0.05–1 *w*/*v*%	Linseed oil	Not reported	Functional coating	[22]
Wood pulp	0.5 *w*/*v*%	Ginger essential oil	Not reported	Active packaging	[23]
Rice straw	0.2–1.0 *w*/*v*%	Cinnamon essential oil	30 d at r.t.	Active substance delivery	[24]
Oil palm empty fruit bunch	0.1–2.0 wt%	Black cumin seed oil	180 d	Active substance delivery	[25]
Kudzu	0.75 wt%	Clove bud oil	Not reported	Food preservation	[26]
Litchi peels	0.3–1.1 wt%	Grape seed oil	30 d at r.t.	Emulsion stabilization	[27]
Mangosteen rind	1.5–4.0 *w*/*v*%	Palm oil	90 d at r.t.	Emulsion stabilization	[28]
Pineapple peel	0.25–0.5 *w*/*v*%	Ginger essential oil	56 d at 25 or 40 °C	Emulsion stabilization	[29]
Camellia oleifera Abel shell	0.2 *w*/*v*%	Camellia oil, Mosla chinensis essential oil	10 d at 4 °C	Emulsion stabilization	[30]
Coir fiber	0.1–0.5 wt%	Sunflower oil	14 d	Emulsion stabilization	[31]
Hazelnut shells	0.5–2 wt%	Sunflower oil	28 d at 25 °C	Emulsion stabilization	[32]
Rice bran	0.1–0.5 *w*/*v*%	Corn oil	28 d at 25 °C	Emulsion stabilization	[33]
Sugarcane bagasse	1–5 wt%	Cooking oil (palm olein)	90 d at r.t.	Emulsion stabilization	[34]
Rosa roxburghii pome	0.5 wt%	Cinnamon essential oil	7 d at r.t.	Active packaging	[35]
Waste jujube kernel	1 wt%	Cinnamon essential oil, Clove essential oil	30 d at 4 °C	Food preservation	[36]
Jujube kernels	0.2–1.0 wt.%	Thymus vulgaris essential oil	Not reported	Active packaging	[37]
Microcrystalline cellulose	0.14 *w*/*v*%	Cinnamon essential oil	Not reported	Active packaging	[38]
Kombucha bacterial cellulose	Not reported	Camellia oil	35 d	Food preservation	[39]

Note: d, day(s); r.t., room temperature.

## 2. Pickering Emulsions Stabilized by Modified CNCs

In existing studies, sulfuric acid hydrolysis is the predominant method for preparing CNCs [40,41]. In this process, cellulose is hydrolyzed with sulfuric acid, introducing negatively charged sulfate ester groups (-OSO_3_H) onto the CNC surface. These groups generate electrostatic repulsion between CNC particles, prevent droplet coalescence, and enable short term emulsion stability [15,42]. However, sulfated CNCs still suffer from poor interfacial adsorption, limited thermal stability, and restricted emulsion types when used as emulsion stabilizers, which limits the functional applications of the resulting emulsions. Sulfated CNCs possess a high absolute zeta potential, which favors electrostatic repulsion between particles, but excessive surface charge can form a strong electrostatic barrier at the oil–water interface, hinder the migration of CNCs from the aqueous phase to the interface, reduce interfacial adsorption, and consequently weaken emulsifying performance [43]. Meanwhile, the introduced sulfate ester groups can catalyze the thermal degradation of cellulose at high temperatures, leading to reduced thermal stability of CNCs [44]. In addition, oil in water (O/W) emulsions contain an aqueous continuous phase and an oil dispersed phase. Because pristine CNCs are highly hydrophilic, they are generally suitable for stabilizing this type of emulsion, whereas stabilizing water in oil (W/O) emulsions remains challenging [45].

To address these issues, chemical modification can regulate the interfacial behavior of CNCs at the molecular level and improve emulsion stability. For example, amination introduces amino groups, which can reduce the negative surface charge of CNCs, increase their water contact angle, and strengthen hydrophobic interactions between CNCs and the oil phase. Esterification grafts hydrophobic segments onto CNCs, thereby significantly enhancing their interfacial stabilizing ability, improving interfacial adsorption, and regulating emulsion type. In terms of functional regulation, these modification strategies can enhance the emulsifying ability of CNCs [46,47,48], endow them with antibacterial, antioxidant, and pH-responsive properties by loading antibacterial substances or covalently introducing functional groups [49,50], and facilitate raw material recovery [51]. These advances provide useful approaches for the sustainable and functional development of Pickering emulsions.

### 2.1. Chemical Modification Methods of CNCs for Stabilizing Pickering Emulsions

A large number of reactive hydroxyl groups are distributed on the surface of CNCs, providing abundant reaction sites for chemical modification. In the cellulose molecular chain, each anhydroglucose unit contains three hydroxyl groups at the C2, C3, and C6 positions, among which the hydroxyl group at C6 shows the highest reactivity. Chemical modification of CNCs is usually carried out through these reactive hydroxyl groups. Common modification methods for CNCs include amination [52], esterification [53], oxidation [54], and graft copolymerization [55]. In recent years, desulfation [56] and etherification [44] of CNCs have also been reported.

**Figure 2:**
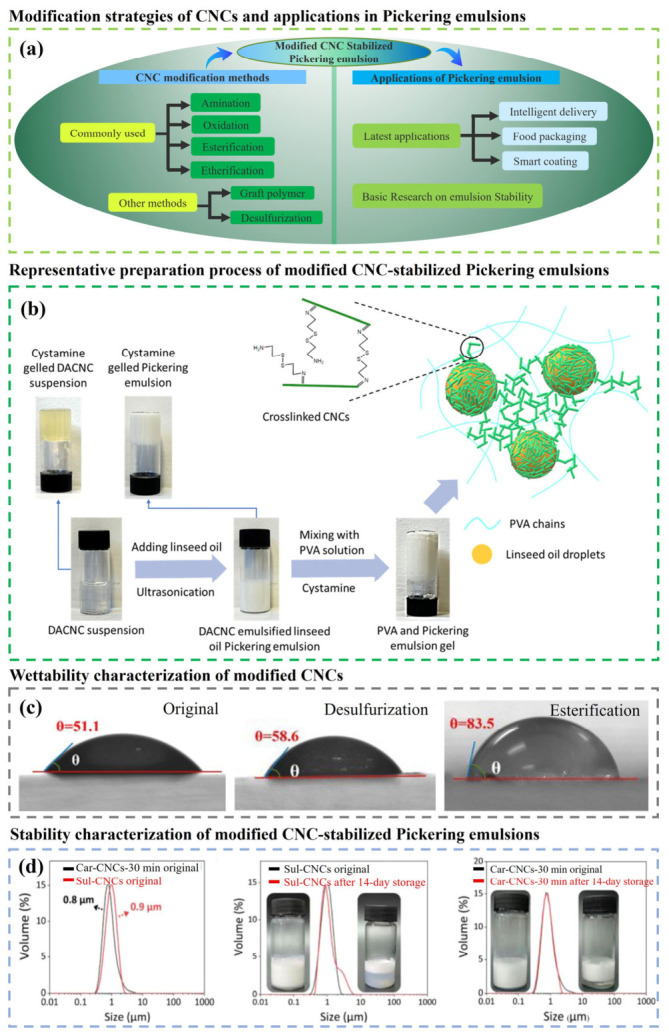
Modification strategies of CNCs and their applications in Pickering emulsions, together with the characterization of modified CNCs and modified CNC-stabilized Pickering emulsions. (**a**) Overview of CNC modification methods and representative applications of Pickering emulsions; (**b**) representative preparation process of modified CNC-stabilized Pickering emulsions (reproduced from [57] under the terms of Creative Commons CC BY 4.0 license, 2024); (**c**) wettability characterization of pristine sulfated CNCs, desulfated CNCs, and esterified CNCs (reproduced from [58] under the terms of Creative Commons CC BY-NC-ND 4.0 license, 2022); (**d**) stability characterization of modified CNC-stabilized Pickering emulsions (reproduced from [44] under the terms of Creative Commons CC BY-NC-ND 4.0 license, 2023).

The stabilization of CNCs at oil–water interfaces mainly relies on the physical barrier formed by these solid particles. When CNCs adsorb onto the surface of oil droplets, their high aspect ratio and rigid structure enable the formation of a dense interfacial layer, which effectively prevents droplet coalescence through steric hindrance. Meanwhile, the negative charges on CNC surfaces generate electrostatic repulsion between droplets and further suppress aggregation. The modification methods described above regulate surface charge, wettability, and related properties of CNCs based on these stabilization mechanisms, thereby optimizing their adsorption and arrangement at oil–water interfaces and improving their ability to stabilize Pickering emulsions. In addition, the active groups or polymer chains introduced by modification facilitate the subsequent functionalization of emulsions, allowing them to show promising application potential in food preservation, active packaging, smart coatings, and controlled-release systems. For food-oriented applications, however, the relevance of these modified systems depends not only on interfacial performance but also on the compatibility of the modifiers, solvents, and residual reaction components with food-contact or oral delivery requirements. The main modification strategies and representative applications are summarized in Figure 2a, and recent studies on Pickering emulsions stabilized by modified CNCs are listed in Table 2.

**Oxidation modification**

Oxidation modification introduces carboxyl or aldehyde groups onto the CNC surface, thereby adjusting the surface charge of the particles, improving their interfacial adsorption and emulsion stability associated with strong hydrophilicity, and providing reactive sites for subsequent crosslinking and grafting reactions, which is beneficial for endowing emulsions with functionality. Common oxidants include periodates [59], APS [43] and TEMPO [60]. As a representative example, Figure 2b shows that oxidized CNCs, namely dialdehyde CNCs, can be further crosslinked with cystamine and used to construct a modified CNC-stabilized Pickering emulsion gel, demonstrating the role of oxidation-derived reactive groups in subsequent crosslinking and emulsion structuring.

Periodate oxidation can selectively cleave the C2–C3 bonds on the CNC surface and introduce aldehyde groups (-CHO). For example, sodium periodate (NaIO_4_) oxidation significantly improves the hydrophobicity of CNCs, increasing the water contact angle from approximately 22° to 56°, thereby strengthening the irreversible adsorption of particles at the oil–water interface. The resulting emulsions remained stable for more than six months of storage [54]. Since aldehyde groups are polar, this increase in apparent hydrophobicity should not be attributed simply to aldehyde introduction. Instead, periodate oxidation disrupts the ordered arrangement of surface hydroxyl groups and can promote intra- or intermolecular hemiacetal formation, thereby reducing surface hydration and increasing the apparent hydrophobicity of CNCs. Although the introduction of aldehyde groups partially reduces the single crystal structure and rigidity of CNCs, it provides highly reactive Schiff base reaction sites for subsequent functionalization. For instance, crosslinking with amino groups in gelatin can be used to prepare edible composite films with good mechanical properties. TEMPO oxidation and APS oxidation can both introduce carboxyl groups (-COOH) onto the CNC surface, enhancing electrostatic repulsion between particles and allowing emulsions to remain stable over a wide pH range. However, these two methods show different emphases in product performance. TEMPO oxidation has the advantages of high catalytic efficiency and mild reaction conditions, whereas the thermal stability of the resulting products decreases markedly with increasing carboxyl content. In contrast, CNCs prepared by APS oxidation show higher crystallinity, better thermal stability, and improved emulsifying performance, although this method is limited by the restricted introduction of surface functional groups as well as insufficient yield and dispersibility [14]. To overcome the limitations of single oxidation methods, combined oxidation modification has become an important direction in recent years. For example, NaIO_4_ pre-oxidation combined with Fenton reagent can simultaneously introduce aldehyde and carboxyl groups onto the CNC surface. This combined method not only increases the yield to more than 58%, but also achieves high crystallinity (83.3%), excellent water dispersibility (transmittance > 85%), and outstanding emulsifying performance (surface coverage of 81.8%) [41]. Fenton reagent uses free radicals generated from hydrogen peroxide decomposition for oxidation and has the advantages of readily available reactants, simple operation, and environmental friendliness. These features are consistent with the requirements of green chemistry and provide a feasible industrial route for the low cost and sustainable preparation of functionalized CNCs.

Oxidation modification introduces carboxyl and aldehyde groups onto the CNC surface. The former improves emulsion stability by regulating surface charge, whereas the latter enables further crosslinking or grafting of functional molecules through controllable reactivity. This allows emulsions to achieve functions such as controlled release of active compounds and pH responsiveness, creating opportunities for the development of smart preservation coatings and active packaging.

**Amination modification**

Amination modification introduces amino groups (-NH_2_) onto the CNC surface, which can regulate surface charge and wettability, improve insufficient interfacial wettability caused by the strong hydrophilicity of CNCs, and enhance emulsion stability. At the same time, antibacterial functional groups can be further introduced through chemical methods such as Schiff base reactions [50] and amidation [42], making aminated CNCs suitable for active compound delivery systems.

Among various amination reagents, PEI has become a commonly used cationic modifier for imparting antibacterial activity to CNCs because of its abundant primary, secondary, and tertiary amine groups and high density of positive charges. PEI grafting is mainly achieved through two routes: Schiff base reactions and amidation reactions, both of which can reverse the surface charge of CNCs. After PEI modification, CNCs generally exhibit a positive zeta potential and enhanced electrostatic adsorption ability, resulting in efficient contact inhibition against negatively charged bacteria [52]. Notably, the protonation and deprotonation behavior of amino groups in PEI and residual carboxyl groups on CNCs under different pH conditions introduces pH responsiveness into the emulsion system. Acidic conditions trigger a hydrophilic transition, leading to emulsion demulsification and release of the encapsulated active compounds. Based on this feature, controlled release of antibacterial agents was achieved at pH 4.0 [49], with the antibacterial rate reaching 100% within 4 h. Thus, PEI amination modification endows CNCs with both antibacterial activity and pH responsiveness, showing considerable potential in responsive antibacterial food packaging and active delivery. Hydrophobic alkylamine modification represents another important direction. Octylamine, cystamine, and other alkylamines can be introduced onto the CNC surface through Schiff base or reductive amination reactions, effectively regulating their amphiphilicity [57]. For example, octylamine grafting increased the water contact angle of CNCs from approximately 30° to 63° and significantly improved their emulsifying performance. Only a 10% concentration was required to encapsulate linseed oil, which was much lower than that required for pristine CNCs [47]. Cystamine grafting can induce the formation of a crosslinked network, leading to emulsion gelation and further enhancing stability.

Amination modification introduces amino and other functional groups onto CNCs and enables the controlled release of active compounds through the pH responsiveness of amino groups. It has developed from a single wettability regulation strategy into a multifunctional approach integrating antibacterial activity, pH responsiveness, and interfacial stability, showing broad application prospects in antibacterial food packaging and related fields.

**Esterification modification**

Esterification modification is a classic and efficient chemical strategy for regulating the surface amphiphilicity of CNCs. By introducing hydrophobic segments onto the CNC surface through esterification reactions, it can compensate for the strong hydrophilicity and weak hydrophobicity of CNCs and significantly enhance the anchoring ability of particles at the oil–water interface. More importantly, esterification modification enables regulation of emulsion type (O/W or W/O) and interfacial film thickness by selecting the acyl chain length and controlling the degree of surface substitution, thereby expanding the application of Pickering emulsions in functional food packaging, cosmetics, and related fields. Esterification is essentially an acylation reaction, and its technical routes can be divided into two main categories: direct esterification between carboxylic acids and surface hydroxyl groups of CNCs under strong acid catalysis [45], and esterification or transesterification using activated carboxylic acid derivatives such as acid anhydrides, acyl chlorides, or vinyl esters under mild conditions [61]. The latter has become the mainstream approach because of its controllable reaction conditions, fewer side reactions, and high grafting efficiency.

Among acid anhydride-based esterification modifications, dodecenyl succinic anhydride (DDSA) and octenyl succinic anhydride are two representative hydrophobic modifiers. After DDSA modification, the water contact angle of CNCs can increase to approximately 74°, indicating markedly enhanced affinity for the oil–water interface. The stabilized emulsions show both oxygen and moisture barrier properties, and the resulting starch based edible films can effectively maintain the freshness of perishable fruits [53]. Octenyl succinic anhydride modification can achieve efficient grafting on both crystalline forms of CNCs, namely CNC I and CNC II. The stabilized Pickering emulsions maintained uniform droplet sizes after 28 days of storage, demonstrating excellent stability [15]. Compared with the acid anhydride method, the acyl chloride method uses more reactive reagents, such as cinnamoyl chloride, for esterification and shows more pronounced advantages in hydrophobic modification efficiency. Studies have confirmed that esterification increases the water contact angle of CNCs more effectively than desulfation treatment, with the contact angle increasing from 51.1° for pristine CNCs to 58.6° after desulfation and 83.5° after esterification (Figure 2c), indicating stronger hydrophobicity and improved suitability for application scenarios requiring highly hydrophobic interfaces [58]. Transesterification modification and emulsion type regulation represent more advanced applications of esterification modification. By systematically changing the length of the introduced acyl chains and the degree of surface substitution, a controllable transition from O/W to W/O emulsions can be achieved [62]. Studies have shown that short-chain acylated CNCs (C2–C6) tend to stabilize O/W emulsions, whereas long-chain acylated CNCs (C ≥ 8) can induce an O/W-to-W/O transition at higher surface substitution. In one reported system, this transition occurred when the degree of substitution exceeded approximately 0.25, so this value should be regarded as system-specific rather than universal. Therefore, esterification can be used to tune CNC wettability and emulsion type, but its effect depends on the specific formulation and emulsification conditions.

**Graft copolymerization**

Graft copolymerization is a surface engineering strategy for imparting multifunctionality to CNCs. Covalently introducing polymer segments with specific functions onto the CNCs surface can simultaneously achieve multiple objectives: the steric hindrance effect of polymer chains markedly improves particle dispersion stability, hydrophobic segments enhance amphiphilicity and strengthen interfacial anchoring, and stimuli responsive segments endow emulsions with intelligent sensing and response behavior toward environmental changes. Compared with single functional group modifications such as amination or esterification, graft copolymerization overcomes the limitations of small molecule modification in functional design and opens a new route for constructing multifunctional Pickering emulsion delivery systems.

For amphiphilicity regulation, both ring opening polymerization and RAFT polymerization can effectively improve the hydrophobicity of CNCs. For example, amino terminated polyphosphate can first be synthesized through ring opening polymerization and then grafted onto the carboxyl groups of TEMPO oxidized CNCs through amidation, increasing the water contact angle from approximately 19.5° to 69.0° and markedly improving hydrophobicity [63]. RAFT polymerization can produce amphiphilic block copolymers with more refined structures. After epoxy ring opening grafting, the surface tension of modified CNCs can decrease to 44 mN/m, showing excellent oil–water amphiphilicity and interfacial activity [55]. Introducing intelligent responsive functions represents a higher level of graft copolymerization modification. Coumarin is a typical functional group capable of photodimerization and photocleavage, and its introduction into CNCs can enable light responsive self-healing functions. Amphiphilic block copolymers containing coumarin monomers can be synthesized by RAFT polymerization and then grafted onto the CNC surface through epoxy ring opening, thereby enhancing CNC amphiphilicity and imparting light responsiveness [64]. The water contact angle of the modified CNCs can increase sharply to 81°, and the droplet size of the stabilized Pickering emulsion can be reduced to 254 nm, much smaller than the micrometer scale size obtained with pristine CNCs. These results fully demonstrate the unique advantages of graft copolymerization in improving emulsion stability and functionality. These findings indicate that graft copolymerization can regulate CNC amphiphilicity and interfacial adsorption by introducing polymer segments with tailored hydrophilic–hydrophobic balance, thereby providing a useful surface-engineering route for improving the stability and functional performance of CNC-based Pickering emulsions.

**Other modification methods**

In addition to the mainstream modification routes described above, including oxidation, amination, esterification, and graft copolymerization, precise regulation of CNC surface charge has also developed into an important modification strategy. CNCs prepared by sulfuric acid hydrolysis contain sulfate groups on their surfaces, which provide negative charge and electrostatic stability. However, a higher surface charge density is not always beneficial. Insufficient charge can easily lead to particle aggregation, whereas excessive charge forms a strong electrostatic barrier that hinders effective adsorption of particles at the oil–water interface. Therefore, for CNCs with different initial charge states, researchers have developed two modification methods with opposite directions: desulfation and etherification, such as carboxymethylation. Both methods regulate surface charge as the entry point, but follow opposite routes and ultimately achieve long term emulsion stability.

Desulfation modification is suitable for CNCs with excessive surface sulfate ester groups. Although such CNCs can prevent interparticle aggregation through strong electrostatic repulsion, the overly strong charge barrier severely suppresses their irreversible adsorption at the oil–water interface, resulting in interfacial coverage often below 50% and reduced emulsifying performance. Desulfation partially removes sulfate ester groups from the CNCs surface through acid hydrolysis [56] or alkaline hydrolysis [58] moderately reducing the surface charge density. This reduces the hindrance of electrostatic repulsion to interfacial adsorption while retaining sufficient charge to maintain basic repulsion between particles and prevent emulsion coalescence, thereby promoting CNC anchoring at the oil–water interface and improving emulsifying performance. Etherification modification is suitable for CNCs with relatively low surface charge. Because electrostatic repulsion is insufficient, these CNCs are prone to irreversible aggregation, leading to emulsion coalescence during storage. Etherification modification introduces high density negatively charged groups, such as carboxymethyl groups (-O-CH_2_-COOH), thereby increasing the absolute zeta potential of CNCs and enhancing electrostatic repulsion. In addition, etherification modification can serve as a pretreatment step. The electrostatic repulsion generated by the introduced negative charges promotes swelling of plant fiber cell walls, exposes more reactive hydroxyl groups, and forms a synergistic effect with subsequent oxidation modification. This synergy not only significantly shortens the oxidation reaction time but also further increases the surface charge density of CNCs, ultimately endowing the emulsions with excellent stability and enabling them to withstand various environmental conditions, including centrifugation and heating [44].

For CNCs with different surface charge states, desulfation or carboxymethylation modification should be selected appropriately to regulate CNC adsorption at the oil–water interface while maintaining sufficient electrostatic repulsion to improve emulsion stability. The key lies in balancing the driving force for interfacial adsorption with electrostatic repulsion between particles.

### 2.2. Stability and Functional Applications of Modified CNC-Based Pickering Emulsions

Modified CNC-based Pickering emulsions have shown marked improvements in stability. Pristine CNCs are susceptible to environmental factors such as pH, salt concentration, and temperature, which can lead to emulsion destabilization. Chemical modification can effectively regulate the surface charge, hydrophilic–hydrophobic balance, and interfacial adsorption ability of CNCs, allowing the emulsion structure to remain intact under complex environmental conditions. For example, Zhang et al. prepared APS-oxidized and TEMPO-oxidized CNCs, namely A-LSCNCs and T-LSCNCs, respectively, and both modified CNCs maintained emulsion stability for 14 days, showing better stability than emulsions stabilized by pristine CNCs [14]. Li et al. prepared modified CNCs by graft copolymerization. Compared with pristine CNCs, modified CNCs-stabilized emulsions exhibited a higher emulsion layer and smaller droplet size, indicating improved emulsifying performance and enhanced emulsion stability [65]. Deep eutectic solvents, which are binary or ternary eutectic mixtures composed of hydrogen bond acceptors and hydrogen bond donors at a certain stoichiometric ratio, have advantages such as low cost, low toxicity, and biodegradability. Wang et al. introduced cationic groups using a deep eutectic solvent to prepare cationic modified CNCs (AH-CNCs). During storage, emulsions stabilized by pristine CNCs and TEMPO-oxidized CNCs (TO-CNCs) showed phase separation within 1 h, whereas obvious separation in AH-CNC-stabilized emulsions occurred only after 7 days [46]. Liu et al. prepared carboxymethylated CNCs (Car-CNCs) through etherification modification and compared their storage stability with that of pristine sulfated CNCs (Sul-CNCs) over 14 days. After storage, Sul-CNC-stabilized emulsions showed droplet aggregation, whereas Car-CNC-stabilized emulsions remained relatively stable throughout the storage period [44].

In practical applications, Pickering emulsions stabilized by pristine CNCs can only maintain short term stability through limited electrostatic repulsion, making it difficult to preserve structural integrity under external environmental influences or to introduce additional functionalities such as antibacterial activity. Their applications in food preservation and related fields are therefore restricted. In contrast, modified CNC-based Pickering emulsions not only show improved stability but can also acquire antibacterial, antioxidant, and other functional properties through chemical modification. The groups introduced by chemical modification provide a structural basis for the subsequent functionalization of emulsions.

**Food preservation**

Fresh fruits, vegetables, and other perishable foods are highly susceptible to microbial spoilage during transportation and storage, leading to substantial food waste every year [66]. Traditional preservation methods mainly rely on chemical preservatives or low temperature cold chains. However, chemical preservatives face consumer resistance because of residue concerns, while cold chains are energy intensive and cannot completely inhibit bacterial growth. Direct incorporation of antibacterial active compounds, such as plant essential oils and antimicrobial peptides, into emulsion coatings can form a protective layer on food surfaces, but these active compounds are prone to volatilization and inactivation, and emulsions stabilized by conventional surfactants tend to undergo phase separation during storage. As emerging emulsion stabilizers, CNCs are derived from renewable cellulose and are intrinsically biodegradable and nontoxic. Modified CNC-based Pickering emulsions make use of the interfacial stabilizing ability of CNCs to effectively encapsulate and slowly release antibacterial substances, while chemical modification can further impart antibacterial activity to the emulsions for food preservation.

Modified CNC-based Pickering emulsions have also been widely explored in active food packaging films and edible coatings. In these systems, surface-modified CNCs improve interfacial adsorption and promote the uniform dispersion of hydrophobic active compounds, such as oregano essential oil and cinnamaldehyde, within film-forming matrices. Compared with pristine CNC-stabilized systems, hydrophobically modified CNCs can enhance emulsion compatibility, film uniformity, and active compound retention, thereby improving barrier, antibacterial, and preservation performance. Accordingly, modified CNC-based Pickering emulsions have been reported to delay the deterioration of fruits, vegetables, and aquatic products, while aminated CNC/starch–beeswax emulsion coatings can form transparent protective layers on fruit surfaces and extend shelf life [53].

## 3. Pickering Emulsions Synergistically Stabilized by CNCs

The principle of synergistic stabilization is fundamentally different from that of chemical modification. For emulsion stabilization, chemical modification improves the wettability of CNCs, regulates the surface charge and electrostatic repulsion of CNC particles, and enhances their anchoring at the oil–water interface, allowing them to form a more stable interfacial layer between oil and water and thereby improving the stability of Pickering emulsions. By contrast, synergistic stabilization involves the introduction of additional co-stabilizing components to stabilize emulsions together with CNCs [67,68,69]. Co-stabilizing components such as proteins, polysaccharides, surfactants, and inorganic nanoparticles can form composite structures with CNCs through noncovalent interactions, significantly improving their interfacial behavior [34,70,71,72] and producing stable synergistic systems. In terms of functional regulation, synergistic stabilization differs from chemical modification, which covalently introduces functional groups. Instead, it incorporates functional molecules through noncovalent interactions, endowing emulsions with diverse functional properties such as antibacterial activity and intelligent responsiveness. Pickering emulsions stabilized by multicomponent synergistic systems can be widely used in food related fields and have become a current research hotspot. At present, the co-stabilizing components commonly used in CNC-based Pickering emulsions for food applications include surfactants/emulsifiers, proteins and their derivatives, carbohydrates and their derivatives, cyclodextrins, polymers, antibacterial functional molecules, inorganic nanomaterials, oils, and essential oils. Table 3 summarizes the co-stabilizing components, stability duration, and functional applications of CNC synergistically stabilized Pickering emulsions reported in recent years.

### 3.1. Mechanisms of Pickering Emulsions Synergistically Stabilized by CNCs

Synergistic stabilization between CNCs and other components is essentially based on noncovalent interactions, such as electrostatic interactions and hydrogen bonding, rather than the formation of covalent bonds as in chemical modification. Through these interactions, CNCs and co-stabilizing components assemble at the oil–water interface and in the continuous phase to form composite structures with specific compositions, dimensions, morphologies, and mechanical properties, thereby achieving emulsion stabilization and functionalization. These composite structures mainly include composite interfacial films and three-dimensional network structures. At the oil–water interface, the abundant hydroxyl and sulfate ester groups on CNC surfaces allow CNCs to interact readily with other particles or molecules through noncovalent interactions, forming composite interfacial films. These composite films improve the insufficient interfacial adsorption of CNCs caused by their strong hydrophilicity and regulate the wettability of composite particles, enabling them to anchor more firmly at the oil–water interface [82]. In the continuous phase, some co-stabilizing components can self-assemble into three-dimensional network structures, which confine oil droplets within the gel skeleton through steric hindrance and further suppress droplet migration and coalescence [90,93]. Notably, although interfacial films and three-dimensional crosslinked networks are located at the oil–water interface and in the continuous phase, respectively, abundant noncovalent interactions such as hydrogen bonding can also exist between these two composite structures, helping to form a more integrated synergistic composite network. Feng et al. investigated the synergistic interaction between gelatin and CNCs and found that, at pH 7, gelatin mainly adsorbed at the oil–water interface to form a gelatin based interfacial film, whereas CNCs were mainly distributed in the continuous phase to form a dense network. Intermolecular hydrogen bonding between the two components enhanced the coupling between the interface and the continuous phase, forming a stronger synergistic network and further improving emulsion stability [94]. When CNCs are used alone to stabilize emulsions, their emulsifying performance is limited by surface hydrophilicity from hydroxyl groups and strong negative charge from sulfate ester groups. Although they can provide electrostatic repulsion and steric hindrance, they are often insufficient to construct an interface continuous phase synergistic composite network. As a result, pristine CNC systems are usually less effective than synergistically stabilized systems in droplet separation, immobilization, and long-term suppression of migration and coalescence [8]. Therefore, the formation and regulation of composite structures are critical for improving emulsion stability and functionality.

**Figure 3:**
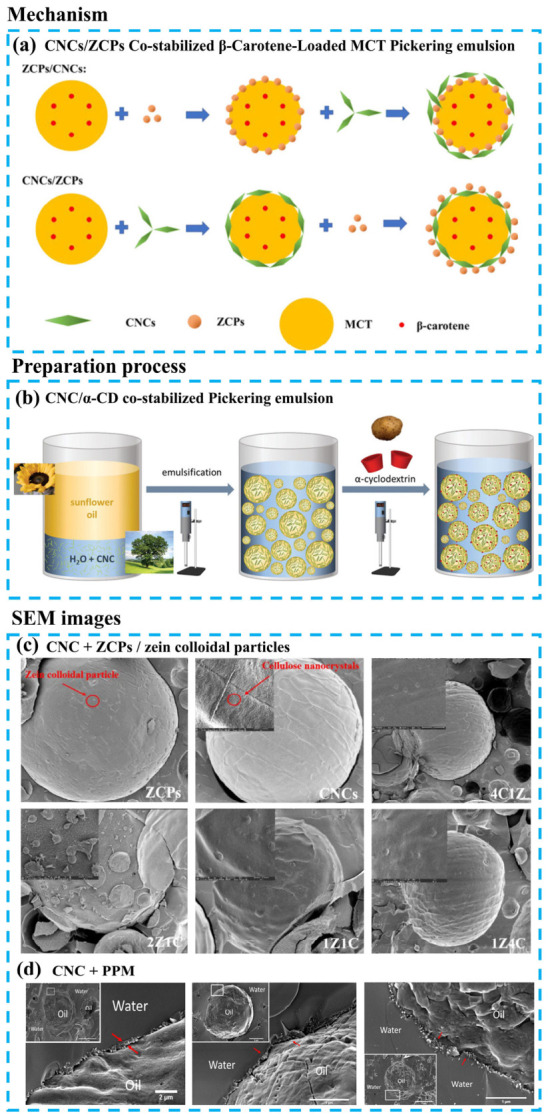
Representative co-stabilization strategies and interfacial microstructures of CNC-based Pickering emulsions with different functional components. (**a**) Schematic illustration of β-carotene-loaded Pickering emulsions co-stabilized by ZCPs and CNCs with different addition sequences (reproduced from [82] with permission from American Chemical Society, 2021); (**b**) CNC/α-cyclodextrin co-stabilized Pickering emulsions prepared with sunflower oil (reproduced from [97] under the terms of Creative Commons CC BY-NC-ND 4.0 license, 2024); (**c**) SEM and Cryo-SEM microstructures of ZCPs/CNC co-stabilized Pickering emulsions with different interfacial compositions (reproduced from [82] with permission from American Chemical Society, 2021); (**d**) Cryo-SEM microstructures of Pickering emulsions co-stabilized by plant protein microgels (PPMs) and CNCs, showing the composite interfacial layer at the oil–water interface (reproduced from [81] with permission from American Chemical Society, 2021). In panels (**c**,**d**), red arrows indicate representative particles and interfacial structures highlighted in the SEM images.

During emulsion stabilization, noncovalent interactions among synergistic components are the prerequisite for the formation of composite structures. Electrostatic interaction is one of the major noncovalent interactions involved in CNC-based synergistic stabilization. Negatively charged CNCs can undergo electrostatic adsorption with positively charged biomacromolecules, such as chitosan and ε-polylysine, to form composite structures. For example, CNCs and chitosan can form a three-dimensional network through electrostatic adsorption and crosslinking [70], which produces a more robust protective layer at the oil–water interface and thereby markedly improves the coalescence resistance and physical stability of emulsions. Co-stabilizing components can regulate the adsorption of CNCs at the oil–water interface and improve the properties of composite structures, thereby enhancing emulsion stability. For example, protein particles and CNCs can form multilayer interfacial structures through noncovalent interactions, increasing the compactness and mechanical strength of the interfacial film (Figure 3a) [82]. This layer-by-layer assembled structure effectively strengthens the irreversible adsorption of particles at the interface and suppresses droplet coalescence, thereby improving emulsion stability. In addition, the representative preparation process shown in Figure 3b illustrates that CNC-based synergistic Pickering emulsions can be constructed through sequential dispersion, emulsification, and incorporation of co-stabilizing components. In addition, the synergistic interaction between two-dimensional nanomaterials, such as GO, and CNCs provides a new approach for interfacial structure regulation [95,96]. The one-dimensional rigid structure of CNCs and the two-dimensional sheet structure of GO can construct a multiscale network through hydrogen bonding, improving interfacial coverage efficiency and film integrity. Compared with systems composed of a single type of particle, this composite structure more readily forms a continuous and compact interfacial layer, effectively preventing droplet coalescence and Ostwald ripening and improving the stability of Pickering emulsions [96].

In terms of emulsion preparation, CNC synergistically stabilized Pickering emulsions are commonly obtained through dispersion, complexation, and emulsification steps. Depending on the synergistic system, ultrasonication, mechanical stirring, or interfacial polymerization can be used to promote interactions among components and interfacial assembly [72]. In addition to the preparation method, the addition ratio and addition sequence of synergistic components also play important roles in regulating emulsion stability and performance. Wei et al. used ZCPs and CNC as synergistic components and prepared a series of Pickering emulsions by layer-by-layer deposition, in which the mass ratio and addition sequence of the two particles were varied for the delivery of β-carotene. CNC particles added first adhered to the droplet surface and formed an inner outer bilayer structure with subsequently added ZCPs particles, which was more favorable for emulsion stability. When the mass ratio of ZCPs to CNC was 1:4, the Pickering emulsion exhibited the best stability, and the retention rate of β-carotene in the emulsion reached 60.23% after storage at 55 °C for 28 days [82].

For functional regulation, the introduction of suitable synergistic components can endow Pickering emulsions with multiple properties, overcome the limitation of traditional methods that mainly load hydrophobic functional molecules in the oil phase, and expand the application scope of Pickering emulsions. For example, the introduction of cationic polymers with antibacterial activity, such as ε-polylysine, can not only enhance interfacial stability but also impart antibacterial functionality to emulsions [72]. Fe_3_O_4_ nanoparticles with superparamagnetic properties can be combined with CNCs to achieve directional migration and demulsification-controlled release of emulsions under a magnetic field [67]. GO with excellent photothermal conversion performance can efficiently absorb light energy and convert it into heat under irradiation, thereby endowing emulsions with solar energy storage and release capability [98]. In addition, composite structures formed by synergistic components can impart other functions to emulsions. For example, Martakov et al. used pseudoboehmite and CNCs to synergistically stabilize Pickering emulsions. When the mass fraction of pseudoboehmite reached 38%, the ζ potential of the composite particles approached zero, weakening electrostatic repulsion between particles and promoting extensive physical crosslinking in the continuous phase to form a three-dimensional gel network throughout the aqueous phase. This gel network showed environmental responsiveness, remaining stable in the stomach and disintegrating in the intestine, thereby precisely controlling the release site of encapsulated substances and enabling targeted controlled release in Pickering emulsions [90].

Overall, an in depth understanding of the mechanisms of CNC synergistically stabilized Pickering emulsions is helpful for precisely regulating key parameters in experimental design, including the type, mass ratio, and addition sequence of synergistic components. By regulating interfacial adsorption behavior and composite structure formation, the wettability of composite particles, compactness of the interfacial film, and strength of the continuous phase network can be optimized, leading to Pickering emulsions with uniform droplet size and excellent stability. In addition, mechanism based synergistic design can endow emulsions with functional properties such as magnetic responsiveness [99], targeted controlled release [90], and photothermal conversion [98], providing theoretical support and practical guidance for the development of multifunctional Pickering emulsions.

### 3.2. Microstructural Characterization of CNC Synergistically Stabilized Pickering Emulsions

The macroscopic stability of Pickering emulsions is closely related to particle adsorption at the oil–water interface, the compactness of the interfacial film, and the microscopic morphology of emulsion droplets. SEM and Cryo-SEM observations provide useful microstructural evidence for understanding how CNCs interact with synergistic components to regulate droplet morphology, interfacial architecture, and continuous-phase networks.

**Droplet size and morphology**

The long-term storage stability of Pickering emulsions depends strongly on droplet size uniformity, sphericity, and dispersibility. Synergistic components can interact with CNCs to regulate droplet formation, suppress coalescence, and improve droplet monodispersity. For example, CNC/lemon seed CNF systems showed improved droplet uniformity and reduced coalescence after lemon seed CNF addition, especially when sufficient LSCNF was introduced to form a composite network around the droplets [86]. Similarly, CNC/GO systems exhibited improved interfacial coverage compared with emulsions stabilized by CNCs or GO alone, which was attributed to the spatially complementary assembly of one-dimensional CNCs and two-dimensional GO sheets through hydrogen bonding [96]. These studies indicate that the combination of CNCs with structurally complementary particles can effectively improve droplet morphology and emulsion stability.

**Composite interfacial film structure**

The strong hydrophilicity and negative charge of pristine CNCs often lead to weak adsorption at the oil–water interface and difficulty in forming a dense interfacial film. Synergistic components can interact with CNCs through electrostatic interactions, hydrogen bonding, or other noncovalent interactions to construct bilayer or multilayer interfacial films, thereby suppressing droplet coalescence. As shown in Figure 3c, in the CNC/ZCPs system, negatively charged CNCs were adsorbed onto the positively charged ZCPs surface through electrostatic interactions, forming a dense bilayer interfacial film with an inner ZCPs layer and an outer CNC coating layer on the oil droplet surface [82]. This bilayer structure enhanced the integrity and mechanical strength of the interfacial film and provided an effective physical barrier against droplet coalescence.

A similar synergistic interfacial adsorption effect has been reported in CNC/plant protein microgel (PPM) systems. As shown in Figure 3d, CNC particles were adsorbed onto the PPM-stabilized oil–water interface, filling the gaps between PPM particles and forming a more continuous composite interfacial film with increased interfacial thickness [81]. This compact interfacial architecture can reduce direct contact between oil droplets, improve the resistance of the interfacial layer to structural damage, and enhance emulsion stability during storage or digestion.

**Three-dimensional crosslinked network structure**

In addition to interfacial film formation, continuous-phase networks also contribute to the stabilization of CNC-based synergistic Pickering emulsions. CNCs alone mainly stabilize emulsions through interfacial adsorption, electrostatic repulsion, and steric hindrance, but their ability to build a strong three-dimensional network is limited. When suitable synergistic components are introduced, CNCs can participate in network formation in the aqueous phase, thereby improving emulsion viscoelasticity, restricting droplet migration, and suppressing flocculation or phase separation. For example, CNC/cyclodextrin systems formed more uniformly dispersed droplets than CNC-stabilized emulsions alone, which was attributed to the formation of continuous CNC/cyclodextrin complexes in the aqueous phase [8]. Therefore, the synergistic stabilization of CNC-based Pickering emulsions is not only governed by interfacial adsorption, but also by the cooperative construction of composite interfacial films and continuous-phase network structures.

### 3.3. Characteristics and Applications of Typical CNC Synergistic Stabilization Systems

The mechanisms of CNC synergistically stabilized Pickering emulsions have been discussed above in terms of composite interfacial films, three-dimensional networks, and noncovalent interactions. On this basis, the structural characteristics and interaction modes of different synergistic components further determine the stability behavior, functional performance, and application direction of the emulsion systems. Existing studies have shown that synergistic systems formed by CNCs with cellulose homologues, inorganic nanosheets, plant protein colloidal particles, and cyclodextrins have demonstrated advantages in emulsion stabilization, active compound delivery, intelligent responsiveness, and food preservation. Therefore, typical synergistic systems can be systematically summarized and compared according to their interfacial characteristics and performance in different application scenarios.

**CNC/cellulose homologue systems**

The advantage of combining cellulose homologues with CNCs lies in their high structural compatibility. Rigid nanocrystals adsorb at the oil–water interface, while flexible fibers form a three dimensional network in the continuous phase, and the two components synergistically improve emulsion stability [100]. Taking the combined system of CNCs and lemon seed derived CNF as an example, their synergy produced well dispersed emulsion droplets and stabilized sunflower oil O/W emulsions with an oil–water ratio of 1:1. No obvious phase separation or flocculation was observed after storage at 4 °C for 15 days, indicating that this system has advantages in suppressing droplet aggregation and maintaining emulsion structure [86]. Similarly, in combined CNC and CNF systems, increasing the CNF content significantly reduced droplet size, while the fibrous network formed in the continuous phase further restricted droplet migration and coalescence, thereby enhancing emulsion stability [101]. Overall, these synergistic systems stabilize emulsions through the combined effects of interfacial adsorption and continuous phase networking, making them suitable for application scenarios with high requirements for system stability and texture.

**CNC/inorganic nanosheet systems**

Unlike cellulose homologues, which mainly contribute to continuous phase network formation, inorganic nanosheets combined with CNCs are characterized by dense interfacial structures and functional expansion. Two-dimensional nanosheets represented by GO can form spatially complementary composite adsorption layers with one-dimensional rod like CNCs at the oil–water interface, thereby enhancing the interfacial barrier effect. Studies have shown that, at appropriate ratios, CNC/GO synergistic systems can significantly reduce droplet size and extend emulsion storage stability. The resulting composite interfacial layer also provides a structural basis for subsequent functions such as photothermal or pH responsiveness [95,96]. Therefore, the significance of this type of system lies not only in improving emulsion stability, but also in using interfacial composite structures to further extend Pickering emulsions toward intelligent responsive materials.

**CNC/plant protein colloidal particle systems**

The synergy between plant protein colloidal particles and CNCs is one of the more systematically studied systems at present. Its main feature is the formation of a thicker and more complete composite interfacial layer on the oil droplet surface, thereby integrating physical stability with the protection of active compounds [1]. For example, when CNCs and ZCPs were combined to stabilize medium chain triglyceride O/W emulsions, the droplet size was significantly reduced, and the retention and release rates of β carotene during storage and digestion were improved, indicating clear advantages in nutrient protection and delivery [82]. In addition, the synergy between CNCs and pea protein microgels can delay emulsion disintegration and lipid release under gastric conditions through the interfacial barrier, making it suitable for functional food design [80,81]. Compared with other systems, this synergistic strategy places greater emphasis on the role of the composite interfacial layer in nutrient delivery and digestion regulation.

**CNC/cyclodextrin systems**

The CNC/cyclodextrin system is characterized by the encapsulation of hydrophobic molecules by cyclodextrins and the three-dimensional network structure formed with CNCs, giving this system unique advantages in encapsulating and protecting volatile active compounds. Studies have shown that cyclodextrins combined with CNCs can effectively stabilize essential oil containing O/W emulsions and improve the retention and antioxidant stability of active compounds during storage [8]. This effect originates partly from the encapsulation of hydrophobic active molecules by cyclodextrins and partly from the steric hindrance and network support formed by CNCs and cyclodextrins. Their synergy can suppress droplet aggregation and the migration of active compounds. Accordingly, CNC/cyclodextrin systems are more suitable for the encapsulation, sustained release, and preservation of active compounds, especially in applications that require both stability and controlled release.

Overall, different CNC synergistic stabilization systems are not simple mixtures of raw materials, but improve emulsion stability and functionality through different pathways, including interfacial adsorption, three-dimensional network support, and the introduction of functional components. The structural characteristics of different synergistic components determine the features and application directions of their corresponding systems. Cellulose homologues are more favorable for enhancing dispersion stability, inorganic nanosheets are more suitable for constructing functional interfaces, plant protein colloidal particles are better suited to nutrient delivery and digestion regulation, and cyclodextrins are more appropriate for active compound encapsulation and sustained release. This classification helps guide the selection of suitable synergistic systems according to target applications.

## 4. Challenges and Prospects

### 4.1. Main Limitations of Modification and Synergistic Stabilization Strategies

Chemical modification and multicomponent synergistic stabilization remain the two most widely used strategies for regulating the performance of CNC-based Pickering emulsions, and a substantial research foundation has been established. Chemical modification regulates the amphiphilicity and interfacial activity of CNCs by introducing specific functional groups onto their surfaces, and is one of the most commonly used methods for improving emulsion stability. However, this strategy still faces several key challenges in further development. First, the modification process is highly sensitive to reaction conditions. Factors such as temperature, reagent concentration, and reaction time often simultaneously affect the aspect ratio, hydroxyl exposure, surface charge, and crystalline structure of CNCs, making it difficult to precisely control their interfacial behavior and emulsion performance [10]. Second, some modification processes may damage the crystalline regions of CNCs, thereby weakening their rigid skeleton and interfacial supporting ability and further affecting the long-term stability and functional performance of emulsions [102]. In addition, the environmental adaptability of modified CNCs is not always satisfactory. For example, oxidized CNCs may undergo damage to the interfacial layer under high temperature conditions, while esterified CNCs may show wettability reversal in high humidity environments [103]. Therefore, although chemical modification can effectively enhance interfacial activity, it still has clear limitations in structural preservation, process controllability, and environmental stability.

The multicomponent synergistic stabilization strategy relies more on the complementary effects between CNCs and proteins, polysaccharides, inorganic particles, or other colloidal components to construct denser or tougher interfacial layers. This strategy shows considerable potential in enhancing emulsion stability and expanding emulsion functionality, but its effectiveness is highly dependent on the specific synergistic system. Electrostatic interactions, hydrogen bonding, and hydrophobic associations between different components are often highly sensitive to the component ratio, and are also easily affected by environmental factors such as pH, ionic strength, and temperature. For example, the synergistic effect between CNCs and proteins may be markedly weakened under acidic conditions, thereby reducing interfacial adsorption and stabilization [13]. In addition, the formation process of composite interfaces is more complex. Excessive synergistic components may hinder the effective adsorption of CNCs at the oil–water interface, or even induce competitive adsorption or interfacial rearrangement, thereby reducing emulsion stability. Furthermore, differences among components during later use or degradation may also lead to reduced integrity of the interfacial layer [104]. These limitations indicate that modification and synergistic stabilization should be evaluated not only by emulsion stability, but also by structural preservation, process controllability, environmental adaptability, and application requirements.

Overall, chemical modification and synergistic stabilization should not be regarded as two competing strategies with fixed advantages or disadvantages, but as complementary routes that need to be selected according to the target application. Chemical modification provides more direct control over CNC wettability, surface charge, interfacial anchoring, and functional group composition, which is useful for designing emulsions with stronger environmental resistance or responsive release behavior. Synergistic stabilization with food-grade proteins, polysaccharides, polyphenols, or other edible colloids is generally more compatible with food formulation requirements, but the resulting interfacial structure is highly dependent on component ratio, pH, ionic strength, and processing conditions. Therefore, the selection of a stabilization strategy should consider food compatibility, scalability, interfacial robustness, safety, and delivery performance together.

### 4.2. Food Compatibility, Migration, and Safety Considerations

For food-related applications, the renewable origin of CNCs should be distinguished from the food-grade status of the final modified particles and emulsions. Pristine CNCs are attractive because of their biomass origin and structural stability, but chemical modification can introduce additional safety and sustainability concerns. Some hydrophobization, grafting, or functionalization routes involve non-food-grade reagents, organic solvents, synthetic polymers, catalysts, or reaction by-products. These systems are useful for understanding how CNC wettability, interfacial adsorption, and release behavior can be regulated, but they should be regarded mainly as model systems unless sufficient purification and safety verification are provided. Therefore, residual solvents, unreacted reagents, modification by-products, migration behavior, digestibility, and toxicological safety should be systematically evaluated before modified CNC-based emulsions are used in food-contact materials or oral delivery systems.

For practical translation, safety evaluation should be linked to the intended use scenario. Food-contact materials require particular attention to migration behavior, residual components, and contact safety, whereas oral delivery systems require further consideration of digestibility, gastrointestinal fate, and toxicological safety. Therefore, model systems involving non-food reagents, organic solvents, or synthetic polymers should not be directly regarded as food-grade emulsions without sufficient purification and safety verification.

### 4.3. Development Trends in Green Regulation Technologies

Given the limitations of modification and synergistic stabilization strategies, new methods centered on green preparation and physical regulation of CNCs have attracted increasing attention in recent years. Unlike approaches that rely on surface chemical modification or complex component assembly, these methods emphasize preserving the intrinsic structure and green attributes of CNCs, while optimizing emulsion performance by regulating the preparation process or dispersion state. Among them, ionic liquid mediated preparation and low frequency ultrasound assisted dispersion are two representative directions. They can optimize the CNC preparation process and adjust the dispersion state of CNCs in solution, respectively, indicating that the preparation of CNC-based Pickering emulsions is moving toward greener and more refined processing.

**Ionic liquid mediated controllable preparation**

Ionic liquid mediated technology provides a new approach for the controllable preparation of CNCs. This method usually uses ionic liquids with low vapor pressure and strong designability as reaction media, selectively acting on the amorphous regions of cellulose and enabling efficient CNC extraction while retaining part of the crystalline regions. Related studies combined the conductor like screening model for real solvents to thermodynamically predict ionic liquid systems formed by different cation and anion combinations. The results showed that the dissolution and dissociation behavior of cellulose in ionic liquids is mainly regulated by hydrogen bonding interactions and intermolecular weak interactions, and shows clear anion dependence [105]. Further experiments found that cellulose dissolving ability and CNC yield are not simply positively correlated, and excessive disruption of intermolecular hydrogen bonds in cellulose may instead inhibit CNC extraction. This indicates that the advantage of ionic liquid mediated preparation is not simply to improve cellulose dissolution, but to achieve a balance between cellulose dissolution and crystalline region preservation.

Compared with traditional chemical modification, the significance of this strategy lies not only in improving CNC yield, but also in achieving more controllable regulation of CNC size, morphology, and crystallinity by tuning the hydrogen bonding parameters and ionic structures of ionic liquids. This provides base particles with clearer origins and more stable structures for subsequent interfacial construction in Pickering emulsions. From this perspective, ionic liquid-mediated technology provides a research basis for exploring the intrinsic relationship between CNC structure and emulsion interfacial behavior. However, for food-related applications, this strategy should be viewed as a controllable preparation method rather than an inherently green or food-grade process, and its practical suitability still depends on ionic liquid selection, purification efficiency, residual solvent control, and safety evaluation.

**Low frequency ultrasound assisted physical dispersion**

Low frequency ultrasound assisted technology mainly regulates the dispersion state of CNC suspensions and represents another typical green physical method. This method relies on local high temperature and high pressure, microjets, and strong shear effects generated by ultrasonic cavitation to effectively dissociate CNC aggregates into better dispersed units, thereby adjusting their distribution at the oil–water interface. Existing studies have shown that, under appropriate frequency, amplitude, and temperature conditions, low frequency ultrasound can disperse CNC aggregates into smaller rod like particles while largely preserving their chemical structure and crystalline characteristics [106].

Compared with chemical modification, low frequency ultrasound does not introduce additional functional groups or external chemicals, which is more favorable for maintaining the green attributes and biocompatibility of CNCs. Compared with multicomponent synergistic stabilization, its advantage lies in directly enhancing the interfacial adsorption ability of CNCs at the particle dispersion level, thereby reducing dependence on additional synergistic components. More importantly, the controllable variables of this method are mainly physical parameters, such as power, time, and temperature, giving it considerable potential in process reproducibility and control. For CNC-based Pickering emulsions, low frequency ultrasound should not be regarded merely as a simple pretreatment step, but may become a key process affecting interfacial adsorption efficiency, droplet size distribution, and environmental stability of emulsions. Therefore, from the perspective of application prospects, ionic liquid mediated preparation and low frequency ultrasound assisted dispersion are expected to form a green pathway from controllable CNC preparation to efficient application. For food-related applications, such green regulation routes are particularly relevant because CNC structural preservation, reduced chemical input, and improved dispersion reproducibility are closely associated with the safety, scalability, and delivery reliability of bioactive-loaded Pickering emulsions.

### 4.4. Application Expansion from Emulsion Stabilization to Bioactive Delivery and Functional Material Construction

As the research focus shifts from emulsion stability to the high-value utilization of emulsion structures, CNC-based Pickering emulsions are increasingly regarded as structure-generating biomaterial platforms. In these systems, droplets, interfacial particle layers, and continuous-phase networks can be further transformed into delivery matrices, coatings, films, or porous solid frameworks. From the perspective of food bioactive delivery, this structural transformation is meaningful because it connects emulsion-based encapsulation and release regulation with the construction of functional carriers and preservation materials. Therefore, the preparation of porous solid materials by emulsion templating and the direct film formation of emulsions for edible preservation systems represent two important directions for extending CNC-based Pickering emulsions beyond liquid dispersion stabilization.

For food bioactive delivery, CNC-based Pickering emulsions mainly contribute through three linked functions: storage protection, release regulation, and bioaccessibility modulation. The dense particle layer at the oil–water interface can reduce the exposure of hydrophobic or volatile bioactives to oxygen, light, and external aqueous phases, thereby improving their retention during processing and storage. At the same time, the interfacial layer, droplet size, oil phase composition, and continuous-phase network can jointly regulate diffusion, digestion, and release behavior. Therefore, future delivery studies should not only report encapsulation efficiency or short-term retention, but also compare long-term storage protection, simulated gastrointestinal release, and final bioaccessibility under food-relevant conditions.

**Construction of porous aerogels by emulsion templating**

Using CNC-based Pickering emulsions as templates to construct porous aerogels has become one of the representative strategies for converting emulsion microstructures into solid functional frameworks. Droplets in emulsions can serve as natural templates and be transformed into porous aerogel structures after freeze drying or supercritical drying. CNCs and synergistic components not only participate in emulsion interfacial stabilization but also constitute the aerogel skeleton, thereby integrating interfacial regulation with solid material construction. Qiao et al. [48] synthesized stable high-internal-phase Pickering emulsions using aminated CNCs and subsequently obtained ultralight CNC aerogels by freeze drying the emulsions. The resulting materials had a density as low as 0.5 mg/cm^3^ and a porosity as high as 99.97%, while also showing excellent thermal insulation performance. These results indicate that Pickering emulsions are not only stable dispersion systems but can also serve as intermediate templates for constructing lightweight and highly porous carrier structures. For food-related delivery and packaging, the relevance of this strategy lies in the possibility of using emulsion-derived porous networks for active compound loading, moisture or gas regulation, adsorption, and sustained release. Future studies should further consider food-contact-compatible components, migration behavior, and preservation or delivery performance under realistic food conditions.

**Direct film formation of emulsions for edible preservation coatings**

Another pathway that is closer to practical industrialization is to directly use CNC-based Pickering emulsions as film forming solutions or edible preservation coatings. In this process, the emulsion not only encapsulates and delivers active compounds, but also directly participates in the formation of the final film or coating structure. The oil–water dispersed structure can be transformed into a composite functional interface, while CNCs simultaneously provide interfacial anchoring and structural reinforcement, thereby improving the mechanical properties, barrier performance, and preservation ability of the coating while stabilizing the emulsion. This strategy avoids the conventional coating preparation process in which the matrix is first constructed and functional components are then additionally introduced, integrating functional component incorporation with structural regulation. The starch beeswax edible emulsion coating prepared by Trinh et al. could effectively block oxygen, maintain food moisture content, and preserve various fruits such as bananas and strawberries. Considering the broad availability, low cost, and edibility of these raw materials, this type of emulsion-based preservation coating is expected to be further extended to wider food systems, including meat, aquatic products, and baked goods [53]. More broadly, the practical translation of CNC-based Pickering emulsions for food bioactive delivery still requires several issues to be addressed. First, food-grade surface modification and co-stabilization strategies should be prioritized to avoid excessive reliance on non-food reagents or organic solvents. Second, emulsion stability should be evaluated using more standardized and comparable indicators, including droplet-size distribution, creaming or sedimentation index, coalescence resistance, zeta potential, rheological stability, and resistance to pH, ionic strength, and thermal processing. Third, future studies should move beyond short-term emulsion formation and pay more attention to long-term storage stability, bioactive retention, gastrointestinal release, bioaccessibility, migration behavior, regulatory status, and scalable processing. These issues are essential for bridging laboratory-scale CNC-based Pickering emulsions with realistic food preservation, active packaging, and nutraceutical delivery applications.

## 5. Conclusions

The stability and functional enhancement of CNC-based Pickering emulsions are mainly governed by the adsorption state of CNCs at the oil–water interface, the structure of the interfacial film, and their interactions with other components. Chemical modification, including oxidation, amination, esterification, graft copolymerization, desulfation, and etherification, regulates the surface charge, hydrophilic-hydrophobic balance, reactivity, and functional group composition of CNCs, thereby improving their interfacial adsorption, emulsion type regulation, and stability under complex environmental conditions. Oxidation and amination provide reactive sites and expand functions such as responsive release and antibacterial activity, while esterification and graft copolymerization improve wettability regulation, hydrophobic segment introduction, and interfacial anchoring. Synergistic stabilization further broadens the structural design of CNC-based Pickering emulsions by combining CNCs with surfactants, proteins, polysaccharides, cyclodextrins, plant polyphenols, inorganic nanomaterials, antibacterial functional molecules, and other components. Through electrostatic adsorption, hydrogen bonding, hydrophobic interactions, and host–guest interactions, these systems can form composite interfacial films or continuous-phase networks, thereby improving interfacial coverage, restricting droplet migration and coalescence, and enhancing emulsion stability during storage, heating, pH variation, salt ion exposure, and digestion.

Together, chemical modification and synergistic stabilization enable CNCs to act as structural and functional units for interfacial construction, active compound loading, and release regulation. Current studies indicate that CNC-based Pickering emulsions have promising potential in food preservation, active packaging, active compound delivery, and other active food systems, particularly for stabilizing, protecting, and regulating the release of food bioactives. Future studies should further clarify the relationships among modification methods, synergistic components, interfacial structures, and final functional performance, especially the effects of composite interfacial film formation, continuous-phase network regulation, and active compound release behavior. Green preparation, safety, environmental adaptability, and stability under practical processing, storage, digestion, and application conditions remain key issues for the practical use of these systems. More systematic mechanistic analysis and application validation will help improve the reliability of CNC-based Pickering emulsions as advanced biomaterial platforms for food-related functional applications.

## Figures and Tables

**Table 2:** Modified CNC-stabilized Pickering emulsions.

Modification of CNCs		Oil Phase	Characteristics of Modified CNCs (Morphology, Size, Zeta Potential)	Application	Main Limitation	Reference
Amination	DA-assisted PEI grafting	Polylactic acid solution	Rod-like; length ~350.8 nm, width ~30.2 nm; zeta potential: +45 mV	Active packaging	Synthetic cationic modifier involved; migration and food-contact safety require further evaluation	[52]
	TEMPO oxidation + PEI grafting	Soybean oil	Rod-like; diameter: 184.68 ± 7.42 nm	Active packaging	PEI grafting improves antimicrobial activity, but particle charge and migration safety are not fully reported	[42]
	TEMPO oxidation + PEI grafting	Oregano essential oil, soybean oil	Rod-like; length ~200 nm, diameter 15–40 nm; zeta potential: +63.07 mV (Mw 25,000)	Active substance delivery	High positive charge and PEI use require further food-contact or oral-delivery safety validation	[49]
Oxidation	NaIO_4_ oxidation	Soybean oil	Short rod-like; length 113.24 ± 6.56 nm (aldehyde content 6.10 ± 0.34 mmol/g)	Active packaging	Aldehyde-containing system; residual aldehydes and migration safety require further evaluation	[54]
	NaIO_4_ oxidation	Sunflower oil	Needle-like; average length 313.72 ± 45.12 nm, width 23.71 ± 7.58 nm; zeta potential: −32.6 mV	Active packaging	Residual oxidants/aldehydes and active compound migration require further evaluation	[59]
	APS oxidation	Sesame oil	Rod-like; diameter 10–60 nm; zeta potential: −33.30 ± 1.14 to −12.63 ± 0.90 mV	Active substance delivery	Bioactivity improved, but gastrointestinal fate and bioaccessibility are not fully evaluated	[43]
	TEMPO oxidation	Peanut oil	CNC: Needle-like, length 170 ± 90 nm, width 3.0 ± 0.5 nm, zeta potential −46 ± 3 mV; CNF: Fibrous, width ~5 nm, length > 1 μm, zeta potential −35 ± 2 mV	Emulsion stabilization	Mainly a model emulsion stabilization study; food application performance requires further validation	[60]
	APS oxidation, TEMPO oxidation	Sunflower oil	APS: Diameter 10–20 nm, length 140–160 nm, zeta potential −31.27 mV; TEMPO: Diameter 26–42 nm, length 340–380 nm, zeta potential −55.67 mV	Emulsion stabilization	Food-derived raw material and sunflower oil are used, but application validation remains limited	[14]
	Octenyl succinic anhydride grafting	Canola oil	CNC I: Rod-like, length 220.5 ± 13.0 nm, diameter 13.0 ± 2.9 nm, zeta potential −35.46 mV; CNC II: Rod-like, length 66.7 ± 13.3 nm, diameter 6.5 ± 1.1 nm, zeta potential −32.89 mV	Food preservation	Food preservation demonstrated, but broader storage, release, and safety evaluation remains limited	[15]
	DDSA grafting	Beeswax	Rod-like; zeta potential: −16.76 mV (for 20 wt% DDSA)	Edible coating	Food-contact relevant coating; migration, sensory effects, and long-term safety require further evaluation	[53]

Note: APS: ammonium persulfate, CNF: cellulose nanofibril, DA: dopamine, DDSA: dodecyl succinic anhydride, PEI: polyethyleneimine, TEMPO: 2,2,6,6-tetramethylpiperidine-1-oxyl, VBMC: 7-(4-vinylbenzyloxy)-4-methylcoumarin.

**Table 3:** Synergistic components and properties of CNC-stabilized emulsion.

Co-Stabilizers		Oil Phase	Representative Stability Indicator or Condition	Application	Food Relevance/System Type	Reference
	Lauric arginate	Soybean oil	Storage stability: 20 d at 55 °C (accelerated test)	Emulsion stabilization	Direct food-relevant	[73]
	Polyglycerol polyricinoleate	Soybean oil	Storage stability: Evaluated over 7 d	Active substance delivery	Oral-delivery relevant	[74]
Proteins & Derivatives	Sodium caseinate	Palm oil	Stability: Evaluated by creaming index and rheology over storage	Emulsion stabilization	Direct food-relevant	[28]
	Lactoferrin	Soybean oil	Storage stability: 30 d (for physical and oxidation stability assessment)	Emulsion stabilization	Oral-delivery relevant	[75]
	Soy protein isolate	Palm oil	Stability: Evaluated over 30 d at 25 °C	Active packaging	Food-contact relevant	[68]
	Peanut protein isolate	Rapeseed oil	Stability: EI > 92% after 1 month storage	Emulsion stabilization	Direct food-relevant	[71]
	β-lactoglobulin	Corn oil	Storage stability: 28 d at 4 °C (for emulsions with >0.8 wt% complexes)	Emulsion stabilization	Direct food-relevant	[76]
	Zein nanoparticles	Corn oil	Thermal stability: 95 °C for 30 min; Storage stability: 30 d at room temperature	Emulsion stabilization	Direct food-relevant	[77]
	Zein colloidal particles (ZCPs)	Cinnamon essential oil	Storage stability: 20 d; Thermal stability: 40, 60, 80 °C for 60 min	Active packaging	Food-contact relevant	[78]
	Gelatin	Soybean oil	Storage stability: Evaluated over 15 d at 4 °C	Emulsion stabilization	Direct food-relevant	[79]
	Pea protein microgels	Sunflower oil	Stability: Evaluated during in vitro gastrointestinal digestion	Emulsion stabilization	Oral-delivery relevant	[80]
	Pea protein microgels	Sunflower oil	Stability: Evaluated over 150 min during in vitro gastric digestion.	Active substance delivery	Oral-delivery relevant	[81]
	ZCPs	Medium chain triglycerides	Storage stability: β-carotene retention evaluated over 28 d at 55 °C	Bioactive delivery	Oral-delivery relevant	[82]
	Peanut protein isolate	Rapeseed oil	Storage stability: Evaluated over 30 d	Active substance delivery	Oral-delivery relevant	[83]
Polysaccharides & Derivatives	Chitosan	Camellia oil	Stability: High resistance to pH (2–12), temperature (4–60 °C), ionic strength (0–1000 mM)	Emulsion stabilization	Direct food-relevant	[70]
	Carboxymethyl cellulose sodium	Clove oil	Stability: Not reported	Active substance delivery	Direct food-relevant	[84]
	α-cyclodextrin, β-cyclodextrin, γ-cyclodextrin	Mosla chinensis essential oil/Camellia oil	Storage stability evaluated over 30 d	Active substance delivery	Direct food-relevant	[8]
	Tannic acid	Corn oil	Storage stability: Evaluated over 2 months at 25 °C	Active packaging	Direct food-relevant	[85]
	CNF	Sunflower oil	Storage stability: 15 d at 4 °C	Emulsion stabilization	Direct food-relevant	[86]
	Tannic acid	Sunflower oil	Storage stability: 30 d at 4 °C	Active substance delivery	Direct food-relevant	[87]
	Octenyl succinic anhydride -modified starch, hydroxypropyl -β-cyclodextrin	Linalool	Storage stability: Creaming Index evaluated over 7 d	Emulsion stabilization	Direct food-relevant	[88]
	Tannic acid	Litsea cubeba essential oil	Storage stability: Not reported	Active packaging	Food-contact relevant	[89]
Inorganic particles	Pseudoboehmite	Olive oil	Storage stability: Stable emulsion fraction > 90% after 1 month (for samples with 9–56% pseudoboehmite)	Active substance delivery	Oral-delivery relevant	[90]
Oils/Essential Oils	Lauric acid	Canola oil	Stability: Evaluated throughout in vitro GIT phases	Lipid digestion and nutrient release	Oral-delivery relevant	[91]
	Ylang-ylang oil	Ylang-ylang oil	Emulsion stability: Evaluated over 15 d	Active packaging	Food-contact relevant	[92]

Note: EI: emulsification index, GIT: gastrointestinal tract.

## Data Availability

No new data were created or analyzed in this study. Data sharing is not applicable to this article.
